# A Survey of Congenital Heart Disease and Other Organic Malformations Associated with Different Types of Orofacial Clefts in Eastern China

**DOI:** 10.1371/journal.pone.0054726

**Published:** 2013-01-21

**Authors:** Ting Sun, Hua Tian, Changqian Wang, Ping Yin, Yaqin Zhu, Xianghua Chen, Zhengde Tang

**Affiliations:** 1 Department of Cardiology, Shanghai Ninth People's Hospital, Shanghai JiaoTong University School of Medicine, Shanghai, P.R. China; 2 State Key Laboratory of Oncogenes and Related Genes, Shanghai Cancer Institute, Renji Hospital, Shanghai Jiaotong University School of Medicine, Shanghai, P.R. China; Pennington Biomedical Research Center/LSU, United States of America

## Abstract

**Background:**

A high incidence of orofacial clefts is reported in China, but no data has shown the relation between cleft types and the incidence of other defects so far. The aim of this study is to assess the incidence of congenital heart diseases and other organic defects associated with different types of orofacial clefts.

**Methodology and Principal Findings:**

All children with orofacial clefts, which were sought out from the Health Information System of Shanghai Ninth People's Hospital between 1^st^ Jan 2009 and 30^th^ Dec 2011, were enrolled in this study. All subjects underwent a thorough examination and grouped by the cleft phenotype. The numbers and types of other organic defects were recorded and analyzed statistically using SPSS 17.0. Of 2180 cases reported as having orofacial clefts, 657 (30.1%) had other congenital abnormalities, which were significantly more common in cleft palate (47.9% (329/687)) than that in cleft lip (10.6% (80/755)) or cleft lip and palate (33.6% (248/738)) (P<0.01). In subgroups, unilateral cleft lip and palate had a statistically higher incidence of associated abnormalities than bilateral cleft lip and palate (P<0.01). The most common malformation was congenital heart disease, which counted 45.1% (296/657) of all malformations. Disorders of the central nervous system (14.3%(94/657)) and Skeletal anomalies (13.1%(86/657)) were also frequently associated. Additionally, the most common defect in heart was atrial septal defect, which was 39.7% (118/296) of all congenital heart diseases.

**Conclusions and Significance:**

As the high incidence of heart defects and other organic abnormalities in the children with cleft palate in Eastern China, special attention should be paid to them and echocardiography should be a proposed examination in the evaluation of children with cleft palate before any surgical correction being executed.

## Introduction

According to anatomical, genetic and embryological findings, orofacial clefts (OCs) are commonly divided into isolated cleft lip (CL), isolated cleft palate (CP), and cleft lip and palate (CLP). OCs are often associated with other congenital abnormalities or organ defects. In the literature there was a considerable controversy on the correlation between associated malformations and types of OCs around the world. Vallino-Napoli reported that one third of the 2022 oral clefts patients had other birth defects and the prevalence rate of CL/P was higher than that of CP from 1983 to 2000 in Victoria, Australia [1]. Beriaghi reported that 32.2% of all cleft patients had other associated congenital malformations, which were more common in CP (38.7%) than in CL/P(26.4%) from 1980 to 2000 in USA [2]. Luijsterburg reported that 10% of all cleft patients showed additional abnormalities of the head and neck area and 13% displayed congenital anomalies of other systems from 1997 to 2006 in Netherlands [3]. Aqrabawi reported that congenital heart disease(CHD) was the most common associated anomalies (47%), followed by skeletal abnormalities (13%) and renal anomalies(10%) among Jordanian infants from 2000 to 2005 [4].

There is also a wide variation in the incidence of CHD in children with OCs in different countries. Shafi et al. [5] found that the most common malformation was CHD, which accounted for 51% of all associated malformations in Pakistan. However, Barbosa et al. [6] reported that the incidence of CHD in patients with CLP was 9.5%, and the presence of CHD did not correlate with types of clefts in Brazil. In China, Liang et al. [7] reported that 56% patients with OCs had additional abnormalities. Central nervous system and skeletal malformations were the most common associated abnormalities, and the overall prevalence of CHD in patients with clefts was 5.4% in Taiwan.

The prevalence of OCs in China is the highest worldwide. Dai L et al. [8] reported that the incidence of OCs was 1.66 per 1000 live births from 1996 to 2005. But it had not been established whether types of clefts were definitely related to CHD or other congenital malformations. The objective of our study was to assess the prevalence and baseline characteristics of associated anomalies in children with OCs from 1^st^ Jan 2008 to 30^th^ Dec 2011 in Eastern China. We investigated the relationship between heart anomalies and cleft types. We also provided these data on some associated pathogenetic factors of OCs with other congenital abnormalities, such as birth weight, maternal age, paternal smoking and maternal influenza.

## Materials and Methods

### Ethics statement

The study was conducted with the approval from the Ethics Committee of Shanghai Ninth People's Hospital, Shanghai JiaoTong University School of Medicine. Written informed consent was obtained from each participant's parents.

### Study population

Between 1^st^ Jan 2009 and 30^th^ Dec 2011, a hospital-based survey on OCs and other malformations was conducted in Shanghai Ninth People's Hospital, the largest cleft care centre in Eeastern China. 2180 children (1219 boys and 961 girls) formed the study, which was nonsyndromic orofacial cleft, excluding Pierre Robin Sequence and other syndromes. The average age was 2.77±2.61 years (range from 2 months to 12 years). The variables registered for the study were age, gender, maternal and paternal condition, birth weight, diagnosis and location of cleft, family history of clefts, and known risk factors for clefts. All children underwent a full clinical examination, plain radiograph, neural axis MRI, ultrasound, echocardiography, intelligence test, and assessment for the presence of other congenital defects both by paediatric cardiologists and plastic surgeons.

### Classification of the clefts

Based on the location of cleft, all children were divided into three groups, which were CL, CP and CLP group. Among them, CL was subdivided into unilateral cleft lip (UCL) and bilateral cleft lip (BCL); CP was subdivided into hard and soft palate cleft (CP(H+S)), soft palate cleft (CP(S)) and submucous cleft palate (SMCP); CLP was also subdivided into unilateral CLP (UCLP) and bilateral CLP (BCLP).

### Statistical analysis

All data were analyzed statistically using SPSS 17.0. Chi-square test was used to compare proportions between two or more groups. *P*<0.05 was considered to be statistically significant.

## Results

### General characteristics of the sample

Of those children with OCs, 755 (35%) were CL, 687 (32%) were CP and 738(34%) were CLP. In CL group, 665 (88%) were UCL and 90 (12%) were BCL. In CP group, 52 (8%) were SMCP, 408 (59%) were CP(S) and 227 (33%) were CP(H+S). In CLP group, 452 (61%) were UCLP and 286 (39%) were BCLP.

The birth prevalence of OCs was varied depending on several factors, including infant sex, birth weight, maternal age, paternal smoking, and maternal influenza. Table 1 provided the percentage distribution of malformations by these factors. Of all cases, 56% were male, 11% had a low birth weight, 12% had a high birth weight, 20% had maternal age over 35, 6% have a maternal influenza history, and 23% have a paternal smoking history. The proportion of malformations was 31.3% in the male group, and 28.7% in the female group, which did not reach the statistical difference. 39.5% had malformations in the low birth weight group, 29.3% in the normal birth weight group and 27.2% in the high birth weight group, which showed that the rate of malformations was much higher in the low birth weight group than other groups (P<0.01). Based on maternal age, the incidence of malformations was 29.2% in low 25 years old group, 28.8% in 25–25 years old group, and 34.9% in over 35 years old group, which had statistically significance (P = 0.05). The rate of malformations was higher in paternal influenza group than that in no paternal influenza group (38.2% vs. 29.7%, P<0.05), and much higher in paternal smoking group than that in no paternal smoking group (37.2% vs. 28.1%, P<0.01). Those data showed the prevalence of malformations was higher among infants with low birth weight, maternal age over 35 years, paternal smoking, or maternal influenza.

**Table 1:** Descriptive characteristics of children with orofacial clefts in Eastern China, 2009–2011.

Subjects' characteristics	OCs (n = 2180)	Malformations		
		No (n = 1523)	Yes (n = 657)	p Value
Gender				0.200
male	1219(56%)	838(68.7%)	381(31.3%)	
female	961(44%)	685(71.3%)	276(28.7%)	
Birth weight(g)				0.003
<2500	233(11%)	141(60.5%)	92(39.5%)	
2500–4000	1675(77%)	1184(70.7%)	491(29.3%)	
>4000	272(12%)	198(72.8%)	74(27.2%)	
Maternal age				0.050
<25	655(30%)	464(70.8%)	191(29.7%)	
25–35	1090(50%)	776(71.2%)	314(28.8%)	
>35	435(20%)	283(65.1%)	152(34.9%)	
Maternal influenza				0.045
no	2057(94%)	1447(70.3%)	610(29.7%)	
yes	123(6%)	76(61.8%)	47(38.2%)	
Paternal smoking				0.000
no	1685(77%)	1212(71.9%)	473(28.1%)	
yes	495(23%)	311(62.8%)	184(37.2%)	

Values are n (%). Comparison between two or more groups by chi-square test.

### Associated malformations

Distributions of OCs by malformations and types were presented in Table 2. Of the 2180 cases, 657(30.1%) cases were found to have other congenital anomalies. Among the three cleft types, 10.6% of the CL children, 47.9% of the CP children and 33.6% of the CLP children had associated other congenital anomalies. The malformations were significantly more common in CP group than those in CL and CLP groups (F = 95.31, P<0.01). In the subgroup of CLP, 173(38.3%) of 452 children with UCLP and 75(26.2%) of 286 children with BCLP had associated malformations. UCLP showed more risk of other congenital abnormalities than BCLP (F = 35.4, P<0.01). In the subgroup of CP, the incidence of associated other congenital anomalies was 15.4% in SMCP, 50.0% in CP(S), 51.5% in CP(H+S), respectively. CP(S) and CP(H+S) had obviously higher incidence of malformations than SMCP statistically (F = 23.9, P<0.01). Although CP(H+S) had slight higher incidence of malformations than CP(S), there was no significant difference between the two groups. In the subgroup of CL, although the incidence rate of malformations in BCL was slightly higher than that in UCL, the difference was not statistically significant.

**Table 2:** Distribution of clefts by malformations and types.

Cleft type	Malformations		p Value*	Organs		p Value^#^
	no	yes		Heart	Others	
CL			0.103			0.719
UCL	599 (90.1%)	66 (9.9%)		27 (40.9%)	39 (59.1%)	
BCL	76 (84.4%)	14 (15.6%)		5 (35.7%)	9 (64.3%)	
Total	675 (89.4%)	80 (10.6%)		32 (40.0%)	48 (60.0%)	
CP			0.000			0.043
SMCP	44 (84.6%)	8 (15.4%)		3 (37.5%)	5 (62.5%)	
CP(S)	204 (50.0%)	204 (50.0%)		79 (38.7%)	125 (61.3%)	
CP(H+S)	110 (48.5%)	117 (51.5%)		62 (53.0%)	55 (47.0%)	
Total	358 (9.9%)	329 (47.9%)		144 (43.8%)	185 (56.2%)	
CLP			0.001			0.453
UCLP	279 (61.7%)	173 (38.3%)		81 (46.8%)	92 (53.2%)	
BCLP	211 (73.8%)	75 (26.2%)		39 (52.0%)	36 (48.0%)	
Total	490 (66.4%)	248 (33.6%)		120 (48.4%)	128 (51.6%)	
Total	1523 (69.9%)	657 (30.1%)		296 (45.1%)	361 (54.9%)	

Values are n (%). *Comparisons of the incidence of congenital malformations among different cleft types in each subgroups by chi-square test. # Comparisons of the incidence of congenital heart defects among different cleft types in each subgroups.

Abbreviations: CL, cleft lip; CP, cleft palate; CLP, cleft lip and palate; UCL, unilateral cleft lip; BCL, bilateral cleft lip; UCLA, unilateral cleft lip and alveolus; BCLA, bilateral cleft lip and alveolus; CP(S), soft palate cleft; CP(H+S), hard and soft palate cleft; SMCP, submucous cleft palate; UCLP, unilateral cleft lip and palate; BCLP, bilateral cleft lip and palate.

Table 3 showed all kinds of the associated other congenital anomalies, which involved many systems, including cardiovascular system, nervous system, digestive system, urinary system, respiratory system, musculoskeletal system, rheumatism immunity system and endocrine system. In CP group, the common additional major defects were CHD (20%), followed by nervous system defects (7.4%), and musculoskeletal defects (5.4%). In CLP and CL groups, musculoskeletal defects were more often than nervous system defects.

**Table 3:** Distribution of malformations by affected organ systems in different type of clefts.

Malformations	CL (755)	CP(687)	CLP(738)	Total
Cardiovascular system	32(3.1%)	144(20.0%)	120 (16.3%)	296(13.6%)
Nervous system	10(1.3%)	51(7.4%)	33(4.5%)	94(4.3%)
musculoskeletal	15(2.0%)	37(5.4%)	34(4.6%)	86(3.9%)
Urinary system	3(0.4%)	24(3.5%)	11(1.5%)	38(1.7%)
Fingers and toes	5(0.7%)	15(2.2%)	14(1.9%)	34(1.5%)
Digestive system	2(0.3%)	17(2.5%)	9(1.2%)	28(1.3%)
Nose	4(0.5%)	6(0.8%)	8(1.1%)	18(0.8%)
Ears	3(0.4%)	9(1.3%)	5(0.7%)	17(0.8%)
Tongue	2(0.3%)	8(1.2%)	6(0.8%)	16(0.7%)
Respiratory system	1(0.1%)	9(1.3%)	4(0.5%)	14(0.6%)
Limbs	1(0.1%)	6(0.8%)	2(0.3%)	9(0.4%)
Eyes	1(0.1%)	2(0.3%)	1(0.1%)	4(0.2%)
others	1(0.1%)	1(0.1%)	1(0.1%)	3(0.1%)
Sum	80(10.6%)	329(47.9%)*	248(33.6%)**	657(30.1%)

Values are n (%). Comparison between two or more groups by chi-square test.

* P<0.01, compared with CLP and CL; **P<0.01, compared with CL.

Abbreviations: CL, cleft lip; CP, cleft palate; CLP, cleft lip and palate.

### Congenital heart disease (CHD)

Among the common associated malformations, CHD was the most common one, which was present in 296 (45.0%) of the 657 cases with associated malformations. The incidence of CHD in CP (20%) was higher than that in CL (3.1%) and CLP (16.3%). From Table 2, in the subgroups of CP, CP(H+S) had a significantly higher incidence of CHD than that of SMCP and CP(S) (P = 0.043). But in the subgroups of CL and CLP, there was no statistical difference in the incidence of CHD between unilateral and bilateral cleft. The types of CHD were shown in Table 4. Atrial septal defect (ASD) was the most common heart defects, which accounted for 39.7% (118/296) of all associated heart malformations. The next most common defect was ventricular septal defect (VSD) (94/296, 31.8%) followed by patent ductus arteriosus (PDA) (34/296, 11.5%) and pulmonary valvular stenosis (PVS) (23/296, 7.8%). In addition, Tetralogy Fallot was also very common in OCs.

**Table 4:** The incidence of congenital heart disease in different type of clefts.

Heart defects	CL (755)	CP(687)	CLP(738)	Total
ASD	16(50.0%)	55(38.2%)	47(39.2%)	118(39.7%)
VSD	12(37.5%)	43(29.9%)	39(32.5%)	94(31.8%)
PDA	2(6.2%)	17(11.8%)	15(12.5%)	34(11.5%)
PVS	1(4.2%)	13(9.0%)	9(7.5%)	23(7.8%)
Tetralogy of Fallot	0	11(7.6%)	8(6.7%)	19(6.4%)
Other defects	1(3.1%)	5(3.5%)	2(1.7%)	8(2.7%)
sum	32(100%)	144(100%)	120(100%)	296(100%)

Abbreviations: ASD, atrial septal defect; VSD, ventricular septal defect; PDA, patent ductus arteriosus; PVS, pulmonary valvular stenosis.

## Discussion

Orofacial cleft, as one of the common birth defects in China, is frequently associated with other congenital anomalies, but the incidence of associated malformations varies widely in the literature, ranging from 1.5% to 63% [9], [10]. Our study showed that 30.1% (657/2180) of the children with OCs were associated with other abnormities. We revealed four factors that may be associated with the incidence of associated malformations, which was birth weight, maternal age, paternal smoking, and maternal influenza. According to our data, mothers over 35 years old were more susceptible to have a baby with other malformations in OCs. Low birth weight infants had an increased risk of having other organic defects. Paternal smoking and influenza during pregnant were also closely related to malformations.

As shown in table 2, the rate of malformations was 47.9% in CP, 10.6% in CL and 33.6% in CLP group, which suggested the children with CP were more likely to have associated malformations than those with CL and CLP. In subgroups, UCL had slightly higher risk of other abnormalities than BCL without statistical difference. UCLP had a statistically higher incidence of associated abnormalities than that of BCLP, to the best of our knowledge, this is the first report on the field in China so far. SMCP had a statistically lower risk of abnormalities than CP(S) and CP(H+S), but no statistical difference was found between CP(S) and CP(H+S) group.

Altunhan H. reported the incidence of congenital anomalies associated with CL/P was 66% in the Konya region, Turkey [11]. Additionally, the incidence of malformations was 32.2% in USA [2], 33.3% in Victoria, Australia [1], and 56% in Taiwan, China [7]. Our study showed the incidence of other congenital abnormalities (30.1%) in OCs was lower than that in other countries, especially in developing countries. This might be due to the following factors: Firstly, children were obtained only from medical records systems, and some infants with OCs were failed to report if they did not choose our hospital. Secondly, with the development of medicine, overall prenatal examination made the serious birth defects rate lower than before. Thirdly, most of children with OCs were presented to hospital for treatment, but few of those from low-income families may be brought into hospital, and very few infants with severe birth defects might not be deserted/abandoned.

The associated congenital anomalies may involve many organs, including brain, heart, lung, liver, spleen, intestines, kidneys, eyes, ears, limbs, fingers, toes, spine, gallbladder, testicle, urethra, bone and muscle. In different countries, there was also a wide variation on the common associated malformations in children with clefts. Rittler et al. [12] reported positive associations of clefts with congenital heart defects, VATER complex (vertebral defects, imperforate anus, tracheoesophageal fistula, and radial and renal dysplasia), and spina bifida. Sárközi et al. [13] reported skeletal anomalies were the most common malformations associated with cleft, followed by disorders of the central nervous system and cardiovascular malformations. Genisca et al. [14] recently found that heart, limb, and other musculoskeletal defects were the most common anomalies associated with orofacial clefts, and central nervous system defects were also common anomalies in cleft palate in USA. In our study, we found the most common defects were heart (13.6%), nervous system (4.3%), and musculoskeletal defects (3.9%). The order of common defects was heart, musculoskeletal, nervous system in CL or CLP, and heart, nervous system, musculoskeletal defects in CP. This difference among different clept groups, has not been described previously in China.

Our study indicated CHD was the most common associated malformation in OCs. The incidence of CHD was 20.0% in CP, 16.3% in CLP, 13.6% in OCs and 3.1% in CL group. Yang et al. [15] reported that the incidence of CHD was 8.2 per 1000 live births in 2000s in China, thus, compared to the general Chinese population, the risk of CHD was about 24 times (20%/0.82%) in CP, 20 times (20%/0.82%) in CLP, 16 times (13.6%/0.82%) in OCs and 4 times (3.1%/0.82%) in CL. Being constant with the common types of CHD in the general population, the most common heart defect in children with orofacial clefts was ASD (118/296, 39.7%), followed by VSD (94/296, 31.8%). In the subgroup of CP, incidence of CHD was 37.5% in SMCP, 38.7% in CP(S) and 53% in CP(H+S), showing some positive relation between heart defects and cleft severity. Han J et al found that retinoic acid, a teratogen induced craniofacial abnormalities, also affected normal heart development by the down-regulation of platelet-derived growth factor C (PDGF-C) [16]. Many studies reported cleft palate was associated with CHD as part of a syndrome [17]. All those data suggested that cleft and CHD might have similar etiologic factors or same induced factors. The children with OCs had a higher incidence of defects in other organs, probably indicating a more widespread embryonic injury.

The authors believe that careful medical check-ups are important for infants with OCs, especially for CP, since they have higher risks of other birth defects. OCs is a disease that can be cured. Before any surgical correction of clefts, all children should have a systematic clinical cardiac assessment as a part of preoperative workup, due to the highest risk of CHD among the associated abnormalities. In our study, most CHD were detected by clinical examination, and some small defects were picked up by echocardiography. Half of the children with heart defects needed treatment in time, and the remainder were under observation. Some children with clefts and mixed heart defects probably needed surgical correction for cardiac defects prior to plastic surgery.

The hospital-based study could not reflect the whole Chinese population, limiting generalizability. However, this is a large sample survey of the cleft phenotype and associated defects. All these data obtained from medical records of our hospital, the largest CLP care centre in Eastern China. Clinical information on all children was reviewed by the plastic surgeon and the pediatrician to assure adherence to case definition and careful classification. Based on those accurate and comprehensive data, our results were reliable and have certain reference value for clinicians.

## Conclusion

Our study showed that the incidence of associated abnormalities was higher in CP than that in CL and CLP. In CL and CLP groups, unilateral clefts had higher risk of other abnormalities than bilateral clefts. In CP group, CP(S) and CP(H+S) had higher risk of other abnormalities than SMCP. The incidence of CHD was higher than other abnormalities, of which ASD and VSD were the most common defects. Echocardiography should be a proposed examination in the assessment of children with cleft palate before any surgical correction being executed. In addition, children with complex CHD and OCs should have firstly needed surgical correction for heart defects.
